# Myrtenal and β-caryophyllene oxide screened from Liquidambaris Fructus suppress NLRP3 inflammasome components in rheumatoid arthritis

**DOI:** 10.1186/s12906-021-03410-2

**Published:** 2021-09-28

**Authors:** Wen-xuan Li, Ping Qian, Yi-tong Guo, Li Gu, Jessore Jurat, Yang Bai, Dong-fang Zhang

**Affiliations:** 1 Department of Pharmacognosy, School of Pharmacy, China Medical University, Shenyang, 110122 Liaoning China; 2 Department of Clinical Pharmacology, School of Pharmacy, China Medical University, Shenyang, 110122 Liaoning China

**Keywords:** Liquidambaris Fructus, Myrtenal, β-Caryophyllene oxide, Rheumatoid arthritis, NLRP3 inflammasome

## Abstract

**Background:**

Liquidambaris Fructus (LF) is the infructescence of *Liquidambar formosana*. In Traditional Chinese Medicine, LF has been used to treat joint pain, a common symptom of arthritis and rheumatism; however, a lack of pharmacological evidence has limited its applications in modern clinics. Therefore, this study aims to explore the protective effect of LF on rheumatoid arthritis (RA) and to identify its active ingredients.

**Methods:**

Rats with adjuvant-induced arthritis (AIA) were divided into 4 groups and administered petroleum ether extract of LF (PEL), ethyl acetate extract of LF (EEL), water extract of LF (WEL), or piroxicam (PIR) respectively for 3 weeks. Two additional groups were used as normal control (NC) and model control (MC) and administered distilled water as a placebo. The clinical scores for arthritis, bone surface, synovial inflammation and cartilage erosion were used to evaluate the therapeutic efficacy of each treatment. The serum IL-1β and TNF-α level and the expression of NLRP3, IL-1β and caspase-1 p20 in the synovial tissue of AIA rats were evaluated by ELISA and Western blot. The active ingredients of LF were investigated using network pharmacology and molecular docking methods, and their inhibition of NLRP3 inflammasome activation was verified in the human rheumatoid arthritis fibroblast-like synovial cells (RA-FLS) model.

**Results:**

PEL could alleviate paw swelling, bone and joint destruction, synovial inflammation and cartilage erosion in the AIA rats, with significantly superior efficacy to that of EEL and WEL. PEL reduced IL-1β and TNF-α serum levels, and attenuated the upregulation of NLRP3, IL-1β and caspase-1 p20 expression in the synovial tissue of AIA rats. Network pharmacology and molecular docking results indicated that myrtenal and β-caryophyllene oxide were the main two active ingredients of PEL, and these two compounds showed significant inhibition on TNF-α, NLRP3, IL-1β and caspase-1 p20 expression in RA-FLS.

**Conclusions:**

Myrtenal and β-caryophyllene oxide screened from PEL could suppress the activation of NLRP3 inflammasome, thereby alleviating RA symptoms.

**Supplementary Information:**

The online version contains supplementary material available at 10.1186/s12906-021-03410-2.

## Background

Rheumatoid arthritis (RA) is an autoimmune disease characterized by chronic inflammation of the synovium. Untreated RA can cause progressive joint destruction, leading to disability, poor quality of life, and increased mortality. Approximately 1% of the world’s population is affected by RA. Onset of disease usually occurs between the ages of 30 and 50, with a higher incidence in women [1–4]. Fibroblast-like synoviocytes (FLS) and inflammatory cells, such as macrophages and T cells, produce proinflammatory cytokines, such as IL-1β and TNF-α, which play key roles in the pathogenesis of RA [5].

In the innate immune system, sentinel immune cells sense the invasion of pathogens and initiate the body’s inflammatory defense response, thereby playing a role in resisting pathogen infection. Nucleotide-binding, oligomerization domain (NOD)-like receptor family, pyrin domain containing 3 (NLRP3) gene polymorphisms have been reported to be associated with susceptibility, disease activity and anti-tumour necrosis factor (TNF) treatment response in RA. RA is a heterogeneous disease entity with a wide range of immunopathological mechanisms and various manifestations, including articular and extra-articular manifestations. In this regard, NLRP3 inflammasome activation is one of the most notable pathological mechanisms that contribute to the pathogenesis of RA [6]. It was reported that the NLRP3 inflammasome is activated in the synovium of RA mice, and treatment with a selective NLRP3 inhibitor can reduce the symptoms of arthritis and cartilage destruction, indicating that targeting the NLRP3 inflammasome with small molecule chemical inhibitors may be a potential therapeutic strategy for the treatment of RA [7–9]. In 2002, the concept of the inflammasome was first proposed by the Tschopp experimental team [10]. Inflammasomes are large multimeric protein complexes, mainly present in innate immune cells that attack pathogens. The three main components of the inflammasome include sensor Nod-like receptors (NLRs), an adaptor protein called ASC and an effector caspase which can hydrolyze and activate pro-IL-1β and pro-IL-18. NLRs are a family of pattern recognition receptors in the cytoplasm that recognize pathogen-related molecular patterns (PAMPs) or damage-related molecular patterns (DAMPs). These patterns are indicative of invading pathogens, prompting NLRs to trigger an innate immune response. Before the inflammasome is activated, the PAMP/DAMP signal triggers the innate immune cells to transcriptionally upregulate the expression of IL-1β and inflammasome sensors. When the triggered cells are stimulated by additional PAMPs/DAMPs, the inflammasome complex assembles and initiates a proteolytic cascade, leading to the hydrolysis and release of IL-1β and IL-18. This usually leads to an inflammatory form of programmed cell death called pyroptosis [10–16].

Liquidambaris Fructus (LF), the infructescence of *Liquidambar formosana*, has been used to treat joint pain and some gynecological diseases in Traditional Chinese Medicine [17]; however, a lack of pharmacological evidence limits the applications of LF in modern clinics. It has been reported that LF shows significant anti-inflammatory effects on xylene-induced ear swelling in mice as well as carrageenan-induced paw edema in rats [18]. LF treatment may decrease the level of inflammatory cytokines in serum, including TNF-α, IL-1β and IL-6, while also increasing the levels of the anti-inflammatory cytokine IL-10. This anti-inflammatory effect is linked to the suppression of COX-2, iNOS and NF-κB p65 [18], indicating LF is a potential crude drug in the treatment of some inflammatory diseases. As joint pain is a common symptom of RA, and it is also the major indication for LF. This study aims to explore the protective effect of LF on RA and to identify its active ingredients while also elucidating the underlying molecular mechanism of action of LF.

## Methods

### Medicinal materials

LF (800 g) was ground and refluxed with 6 times 95% ethanol (*w*/*v*). The extraction process was repeated 3 times and the extracting solution was concentrated under reduced pressure. The residue was dispersed in water and successively extracted with petroleum ether and ethyl acetate to obtain petroleum ether extract of LF (PEL), ethyl acetate extract of LF (EEL) and water extract of LF (WEL). Each extract was concentrated under reduced pressure and dried. The content of β-caryophyllene oxide and myrtenal in PEL were 0.13 and 0.16%, respectively, as detected by high performance liquid chromatography (HPLC).

### Animals

36 Specific Pathogen Free (SPF) male Wistar rats (170–200 g) (Liaoning Changsheng Biotechnology Co., Ltd.) were maintained in the Laboratory Animal Department of China Medical University. The rats were kept at 22 ± 2 °C, 55 ± 5% humidity and a 12-h light/dark cycle with access to food and water ad libitum. The experiment was approved by the Animal Ethics Committee of China Medical University (Approval Number: CMU2019128) before the experiment. The experiment was carried out in strict accordance with the international rules and regulations of laboratory animal ethics.

### Adjuvant-induced arthritis (AIA) animal model

36 rats were divided into 6 groups (*n* = 6). Healthy rats without any treatment were used as the normal control group (NC). Except for NC, all rats were injected intracutaneously with 150 μl Freund’s complete adjuvant (CFA) in the plantar area of the right hind paw on day 0 to establish the AIA rat model as the model control group (MC) [19]. Three different extracts of LF (PEL, EEL and WEL) as well as piroxicam (PIR) were each dissolved in distilled water for administration. Intragastric administration of each treatment was performed starting on the 8th day after CFA injection until the 28th day post-injection. NC and MC were given water (2 ml). The positive control group was given PIR (10 mg/kg/day) as the standard therapy for AIA rats. The other three groups of AIA rats were given PEL (75 mg/kg/day), EEL (100 mg/kg/day) and WEL (130 mg/kg/day), respectively.

### Clinical scoring of AIA

On day 0 of CFA injection, the body weight and the paw volume of the right hind paw were measured. To measure the paw volume, the area 0.2 mm below the ankle joint of each rat’s right hind paw was marked. Then, 18 ml of water was drawn into a 20 ml syringe. The right hind paw of the rat was moved into the 20 ml syringe until the water reached the mark under the ankle. The water in excess of the 18 ml mark was then removed using a 5 ml syringe. The volume of water that was drawn into the 5 ml syringe was equal to the volume of the rat paw. The thickness and circumference of the right hind paw was measured with a vernier caliper and a soft ruler. Body weight, paw volume, paw thickness and paw circumference were further measured on days 7, 12, 16, 20, 24 and 28, and the relative inhibition of paw swelling was calculated. The arthritis index was used to assess the clinical severity of arthritis. The presence of edema and erythema was measured in the ipsilateral and contralateral paws on a scale of 0 to 4 (0 = no swelling, 1 = toe joint swelling, 2 = toe and toe joint swelling, 3 = ankle joint swelling, and 4 = swelling of the whole paw, resulting in immobility) [20]. On the 29th day, the animals were anesthetized with urethane, their serum was collected, the animals were sacrificed by cervical dislocation, and their right hind limbs were taken and fixed in 4% paraformaldehyde.

### Micro-computed tomography (Micro-CT)

Bone destruction was measured using micro-CT. The rats underwent micro-CT scans of their right hind limbs under anesthesia [21]. The procedure was executed using the Skyscan 1276 Micro-CT from Bruker, Germany.

### Hematoxylin-eosin (HE) and safranin O-fast green staining

After decalcification with 10% ethylenediaminetetraacetic acid (EDTA) for 4 weeks, the ankle joints of the hind limbs were embedded in paraffin wax. Ankle joint sections were stained with HE staining. Safranin O-fast green staining was used to evaluate inflammation and cartilage destruction. The following scale was used to evaluate inflammation: 0 = no inflammation; 1 = slightly thickened lining or some infiltration within the lining; 2 = slightly thickened lining and some infiltration within the lining; 3 = thickened lining, a large influx of cells in the lower layer, and an increased number of cells in the synovial space; 4 = profound infiltration of inflammatory cells within the synovium. The following scale was used to evaluate cartilage destruction: 0 = no damage; 1 = minimal corrosion limited to a single spot; 2 = slight to moderate corrosion in a limited area; 3 = wider corrosion range; 4 = widespread damage to cartilage.

### Cytokine detection

Quantification of IL-1β and TNF-α in rat serum and cell culture supernatant was accomplished with human IL-1β (Elabscience, Wuhan, China) and TNF-α (mlbio, Shanghai, China) enzyme-linked immunosorbent assay (ELISA) kits according to the manufacturer’s protocols. Cytokine concentration was evaluated using a microplate reader (Thermo) at 450 nm.

### Western blot

RIPA lysis buffer (PMSF: RIPA = 1:99) supplemented with PMSF (Beyotime, Shanghai, China) was used to extract protein from ankle joint synovial tissue or cell culture. Protein concentration was determined using a bicinchoninic acid (BCA) kit (Beyotime, Shanghai, China). The protein extract was loaded on 10% sodium dodecyl sulfate-polyacrylamide gel electrophoresis (SDS-PAGE) for electrophoresis, and then transferred to a polyvinylidene fluoride (PVDF) membrane (Merck Millipore, Darmstadt, Germany) at 300 mA for 2.5 h. The membrane was blocked with 5% skimmed milk powder (diluted with 0.05% Tween-20 in TBS) at room temperature for 1 h and incubated with primary antibodies overnight at 4 °C, including rabbit anti-NLRP3 (Abcam, UK) (1/1000 dilution), rabbit anti-caspase-1 p20 (Abclonal, China) (1/1000 dilution), rabbit anti-IL-1β (Abclonal, China) (1/1000 dilution) and rabbit anti-glyceraldehyde phosphate phosphate dehydrogenase (GAPDH) (Bioworld, China) (1/10000 dilution). After washing, the membrane was incubated with goat anti-rabbit immunoglobulin (IgG) coupled with horseradish peroxidase (HRP) (Abclonal, China) (1/10000 dilution) at room temperature for 2 h. After washing, the target protein was visualized by detecting the fluorescence generated by enhanced chemiluminescence (ECL) treatment.

### Network pharmacology analysis and molecular docking

Structural information of components in PEL was obtained from the Traditional Chinese Medicine Integrated Database (TCMID, http://www.megabionet.org/tcmid/), the TCM Database@Taiwan (http://tcm.cmu.edu.tw/), the Traditional Chinese Medicine Systems Pharmacology Database (TCMSP) and literature reports [22–24]. Potential targets of these components were predicted using the PharmMapper server (http://lilab.ecust.edu.cn/pharmmapper/) [25]. The top 99 targets of each component acquired from PharmMapper were selected as potential targets in the present study. The different genes associated with RA were gathered from Therapeutic Target Database (TTD) (http://bidd.nus.edu.sg/group/cjttd/) [26] with keywords “Rheumatoid Arthritis”.

A protein-protein interaction (PPI) network was constructed using String [27]. The network visualization software Cytoscape (http://cytoscape.org/) was adopted to visualize the PPI networks [28]. Three topological features of each node in the network were calculated to find the key nodes: “degree,” “node betweenness,” and “closeness.” “Degree” is defined as the number of edges to node i; “node betweenness” represents the number of shortest paths between pairs of nodes that run through node i; “closeness” is the inverse of the sum of the distance from node i to other nodes.

Crystal structures of protein targets were collected from the Protein Data Bank (PDB), and ligands contained in the crystal structures were set as reference ligands. Molecular docking experiments were performed in Molecular Operating Environment (MOE, 2020). Pocket atoms were set as docking sites. For each component-protein target complex, 5 conformations with the lowest binding free energy were obtained. Docking results were analyzed by contrasting the ligand-target interactions of the components with that of the reference ligand, with higher similarity corresponding to better binding affinity.

### Cell culture and treatment

The source of human rheumatoid arthritis fibroblast-like synovial cells (RA-FLS) in this study was from the European Collection of Authenticated Cell Cultures (ECACC), and human synovial tissues were collected from donors with RA. RA-FLS were purchased from Jennio Biotechnology Co., Ltd. Under an inverted phase-contrast microscope, RA-FLS have certain characteristics that distinguish them. FLS are triangular and spindle-shaped, with an oval nucleus, a clear nucleolus in the center of the cell, with an accumulation of secretions surrounding the cells. RA-FLS were cultured in DMEM-H complete medium (Jennio, Guangzhou, China) supplemented with 2% GermClean, L-glutamine, sodium bicarbonate and 15% heat-inactivated FBS (Gibco, Carlsbad, California, USA). Before stimulation, the cells were seeded in a six-well plate and treated with DMEM-H medium containing 10 μmol/L β-caryophyllene oxide (TargetMol, China) and myrtenal (yuanye, Shanghai, China) for 24 h. Cells were stimulated with 10 ng/ml lipopolysaccharide (LPS) (Solarbio Life Science, Beijing, China) for 6 h. Cell lysates and supernatants were collected and used for cytokine detection and Western blot analysis.

### Statistical analysis

All data collected are expressed as mean ± standard error of mean (SEM). SPSS software version 16 was used to analyze the data. Differences were assessed by Dunnett’s multiple comparisons test and one-way or two-way analysis of variance (ANOVA). *P* values less than 0.05 were considered statistically significant.

## Results

### PEL treatment alleviated arthritis in AIA rats

**Fig. 1:**
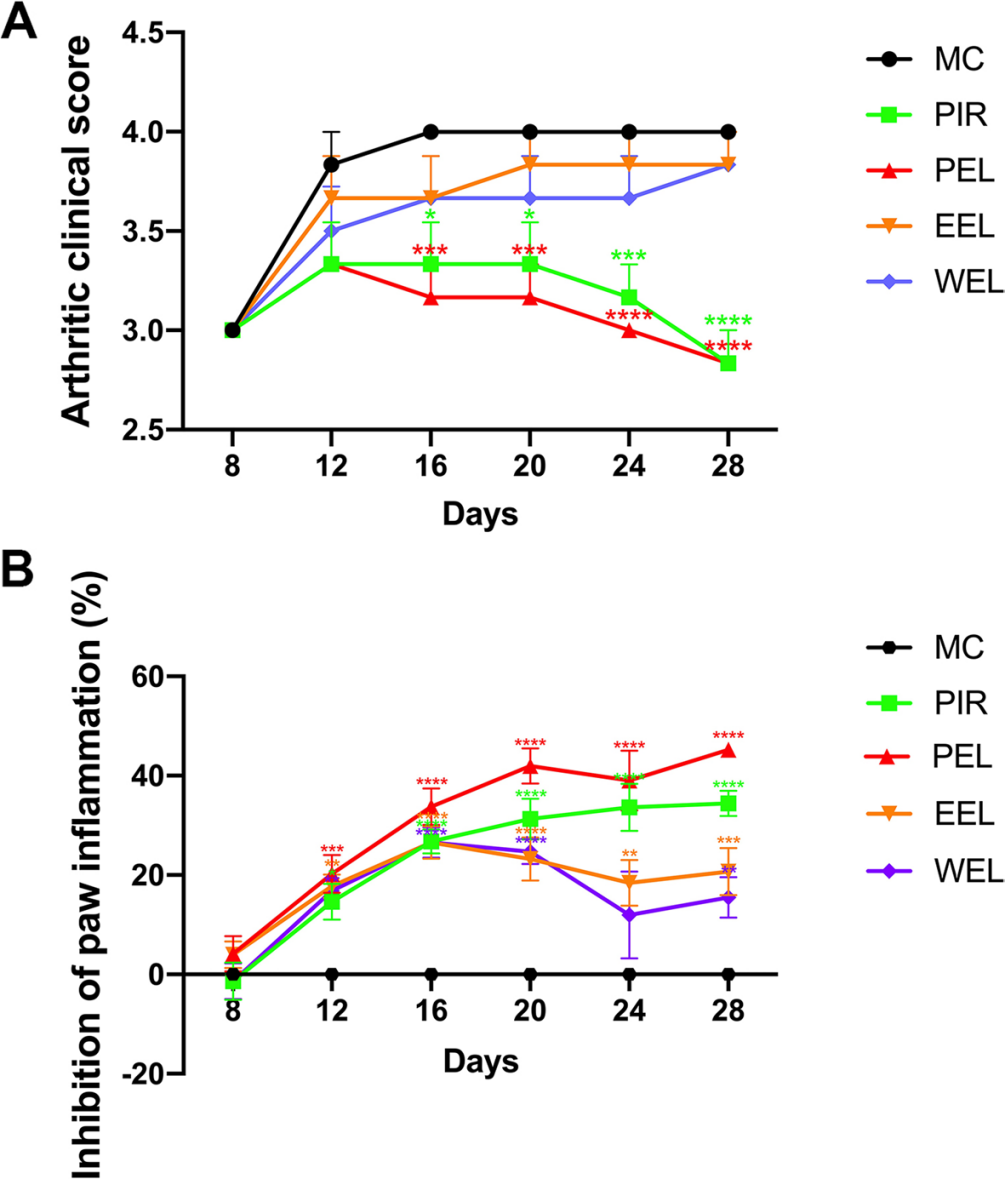
Petroleum ether extract of LF (PEL) alleviated the severity of adjuvant-induced arthritis (AIA), and the efficacy was superior to that of ethyl acetate extract of LF (EEL) and water extract of LF (WEL). **A** Mean clinical scores of AIA rats with different treatments. **B** Inhibition of paw inflammation of AIA rats with different treatments. Paw inhibition rate was calculated as follows: (paw volume before treatment − paw volume after treatment)/paw volume before treatment. Data were recorded as mean ± SEM. Differences were assessed by Dunnett’s multiple comparisons test and two-way ANOVA, *P < 0.05 vs MC, **P < 0.01 vs MC, ***P < 0.001 vs MC, ****P < 0.0001 vs MC. (*n* = 6)

**Fig. 2:**
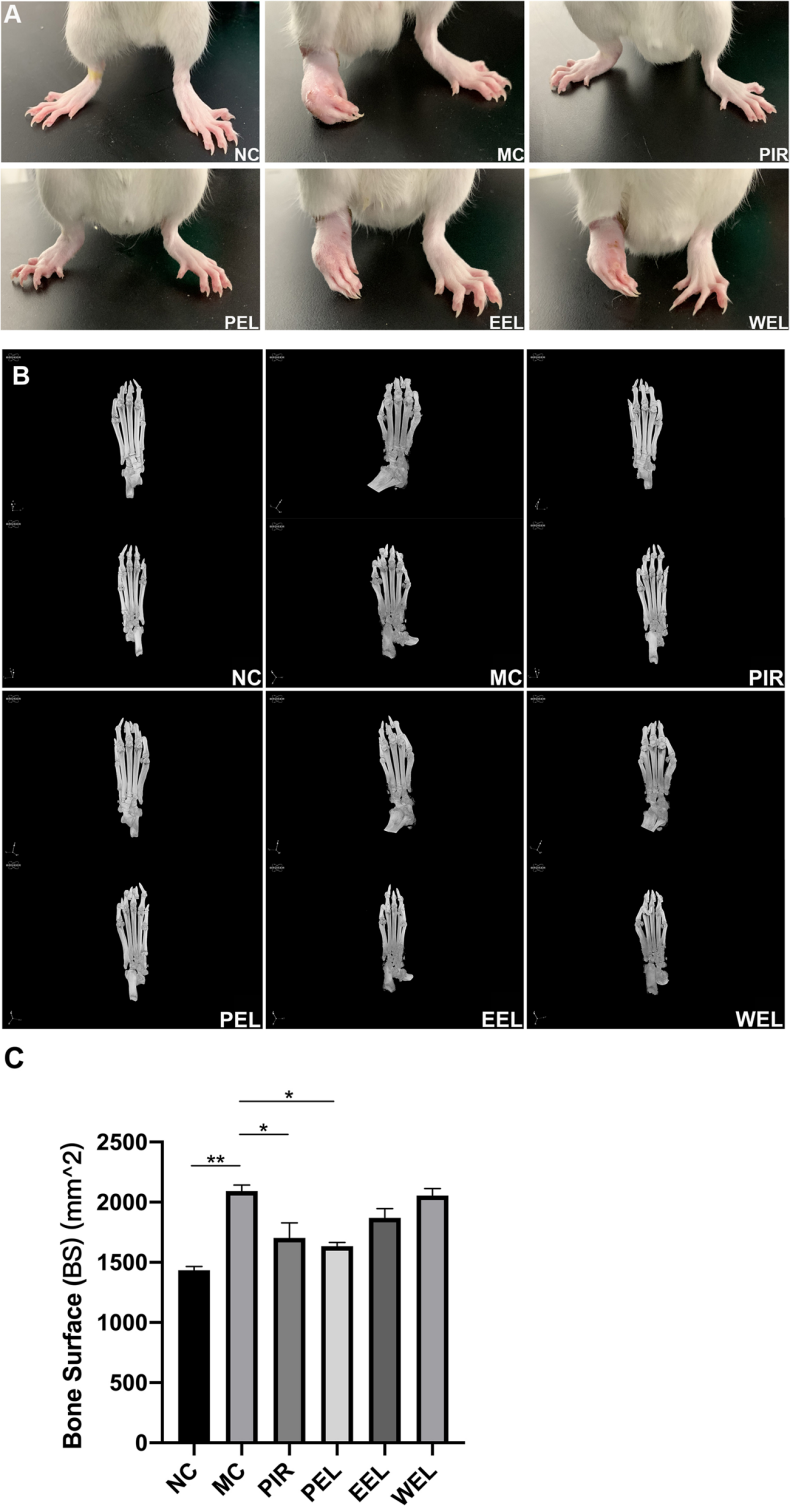
Petroleum ether extract of LF (PEL) treatment significantly alleviated paw swelling and bone erosion in adjuvant-induced arthritis (AIA) rats, and the efficacy was superior to that of ethyl acetate extract of LF (EEL) and water extract of LF (WEL). **A** Macroscopic observation of paws of AIA rats with different treatments. **B** Micro-CT analysis of joints of AIA rats with different treatments under anesthesia using the Skyscan 1276 Micro-CT. **C** Bone surface area calculation of joints of AIA rats with different treatments, where higher bone surface area means more severe bone erosion. Data were recorded as mean ± SEM. Differences were assessed by Dunnett’s multiple comparisons test and one-way ANOVA, *P < 0.05 vs MC, **P < 0.01 vs MC. (*n* = 2)

Beginning on the 8th day after CFA injection, AIA rats were given one of the 3 different extracts of LF, PIR, or water daily via intragastric administration until the 28th day. Results showed that treatment with PEL could improve the clinical arthritic score and paw inflammation of AIA rats from the start of treatment until the end of the study, and the efficacy was superior to that of EEL and WEL (Fig. 1A, B). Compared with NC, untreated AIA rats showed significantly increased foot swelling and surface area, as well as increased bone destruction. Treatment with PEL significantly attenuated the paw swelling of AIA rats (Fig. 2A). The results of micro-CT imaging and bone surface area analysis also consistently showed that the bone and joint destruction of AIA rats were significantly alleviated after the treatment with PEL, but had no significant change after the treatment with EEL or WEL (Fig. 2B, C).

### PEL treatment alleviated synovial inflammation and cartilage erosion in AIA rats

**Fig. 3:**
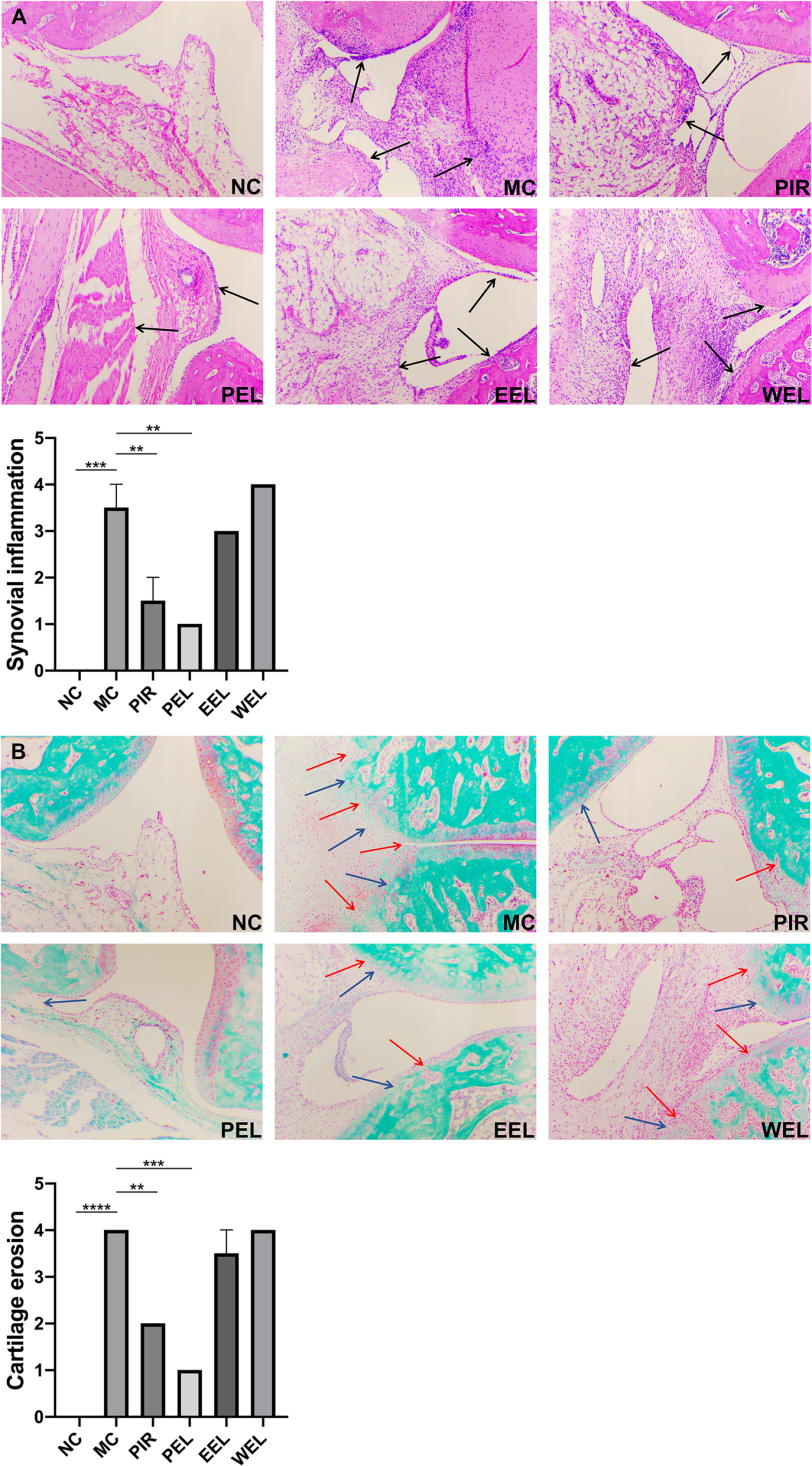
Petroleum ether extract of LF (PEL) treatment significantly alleviated synovial inflammation and cartilage erosion in adjuvant-induced arthritis (AIA) rats. **A** Representative HE staining images and amplification of respective typical lesions (× 100) and semiquantitative score of synovial inflammation. Black arrows point to the infiltration of inflammatory cells or synovial hyperplasia. **B** Representative safranin O-fast green staining images (× 100) and semiquantitative score of cartilage erosion. Red arrows point to areas of cartilage loss. Blue arrows point to areas of bone erosion. Semiquantitative scores of synovial inflammation and cartilage erosion in NC were set to 0. Data were recorded as mean ± SEM. Differences were assessed by Dunnett’s multiple comparisons test and one-way ANOVA, **P < 0.01 vs MC, ***P < 0.001 vs MC, ****P < 0.0001 vs MC. (*n* = 2)

In order to further evaluate the influence of LF on synovial pathology and cartilage destruction, histological evaluation was performed at the end of the study. Compared with NC, significant synovial hyperplasia, pannus formation and inflammatory cell infiltration were observed in the ankle joint of AIA rats. Conversely, only a small amount of local synovial hyperplasia was found after the treatment with PEL (Fig. 3A). The synovium of AIA rats destroyed the bone continuum from both sides and spread to the bone marrow cavity. However, treatment with PEL led to less severe bone destruction (Fig. 3B).

### PEL treatment inhibited the production of IL-1β and TNF-α in AIA rats

**Fig. 4:**
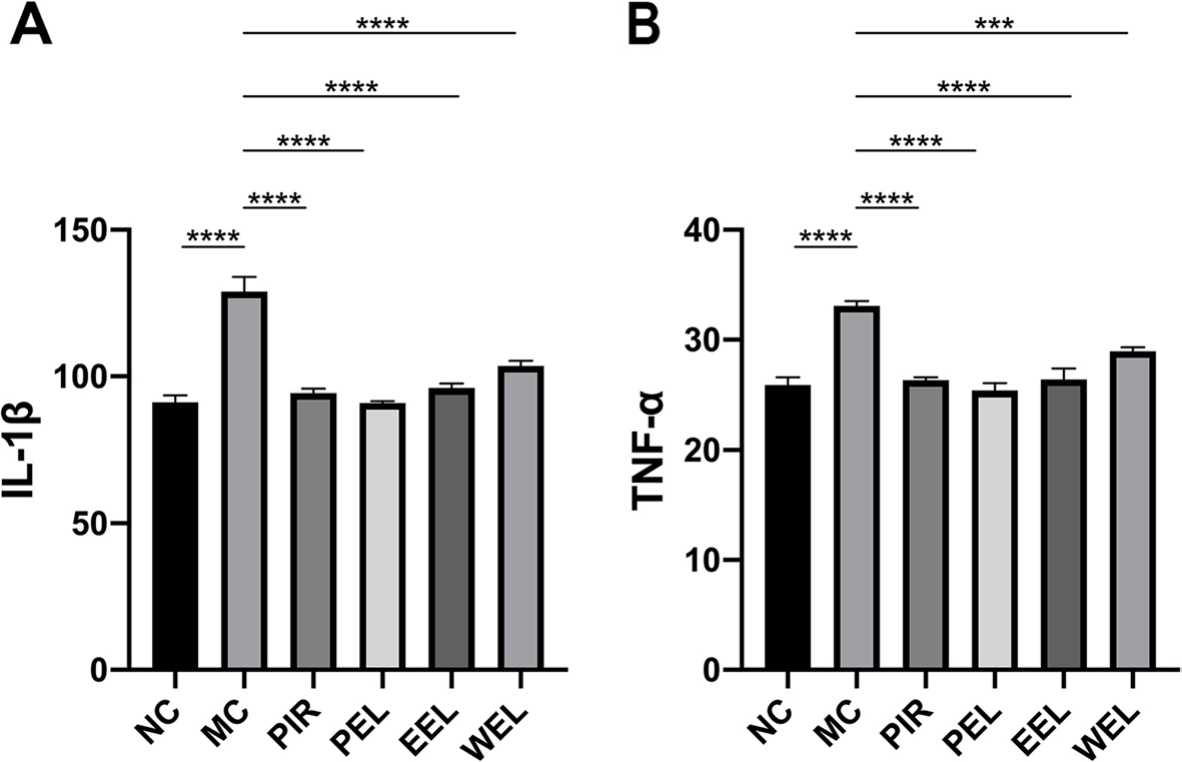
The serum levels of IL-1β and TNF-α of the adjuvant-induced arthritis (AIA) rats were quantified using ELISA. Petroleum ether extract of LF (PEL) treatment significantly reduced the production of IL-1β and TNF-α in the serum of AIA rats, and its efficacy was superior to that of ethyl acetate extract of LF (EEL) and water extract of LF (WEL). Data were recorded as mean ± SEM. Differences were assessed by Dunnett’s multiple comparisons test and one-way ANOVA, ***P < 0.001 vs MC, ****P < 0.0001 vs MC. (*n* = 6)

Similar to the morphological changes and histopathological lesions, the serum levels of inflammatory cytokines IL-1β and TNF-α of the AIA rats also increased significantly compared with NC. After 3 weeks of treatment, the three different extracts of LF all significantly reduced the production of IL-1β in the serum of AIA rats (*P* < 0.0001). However, WEL treatment showed no effects on the serum levels of TNF-α of the AIA rats, and PEL exhibited efficacy superior to that of EEL (Fig. 4).

### PEL inhibited the activation of NLRP3 inflammasome in AIA rats

**Fig. 5:**
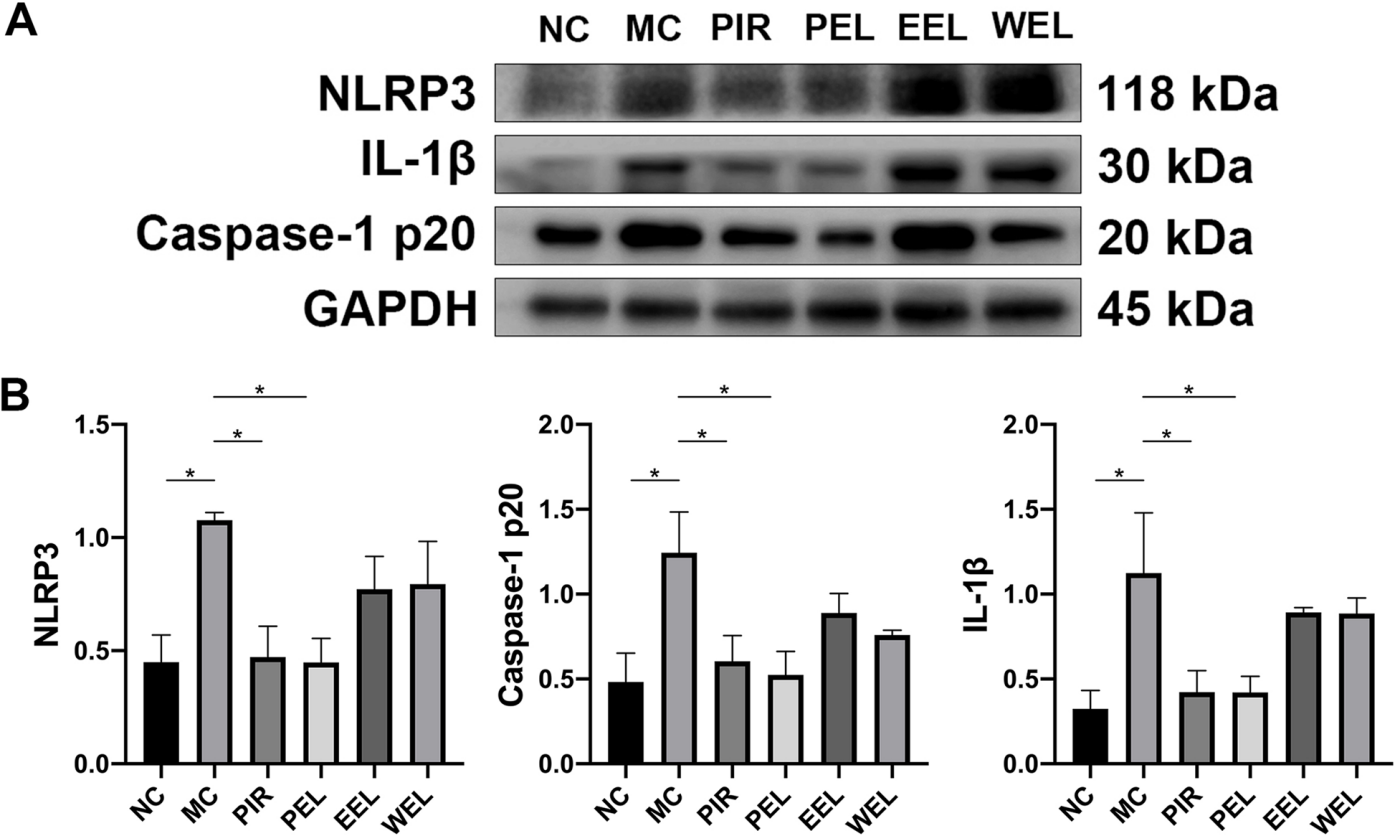
NLRP3 inflammasome pathway was activated in the ankle joint synovial tissue of the adjuvant-induced arthritis (AIA) rats. Treatment with petroleum ether extract of LF (PEL) significantly suppressed the expression of IL-1β, caspase-1 p20 and NLRP3, whereas treatment with ethyl acetate extract of LF (EEL) and water extract of LF (WEL) did not show significant improvement. Data were recorded as mean ± SEM. Differences were assessed by Dunnett’s multiple comparisons test and one-way ANOVA, *P < 0.05 vs MC. (*n* = 3)

Because the activation of the NLRP3 inflammasome could induce the overproduction of IL-1β, resulting in inflammation and arthritis, we evaluated the expression of NLRP3, caspase-1 p20 (a functional caspase-1 subunit) and IL-1β in the ankle joint synovial tissue by Western blot. AIA rats displayed significantly higher expression of all three proteins, but expression was significantly decreased by treatment with PEL. However, treatment with EEL or WEL did not show significant improvement in NLRP3 inflammasome activation in the ankle joint synovial tissue of the AIA rats (Fig. 5).

### Network pharmacology analysis

**Fig. 6:**
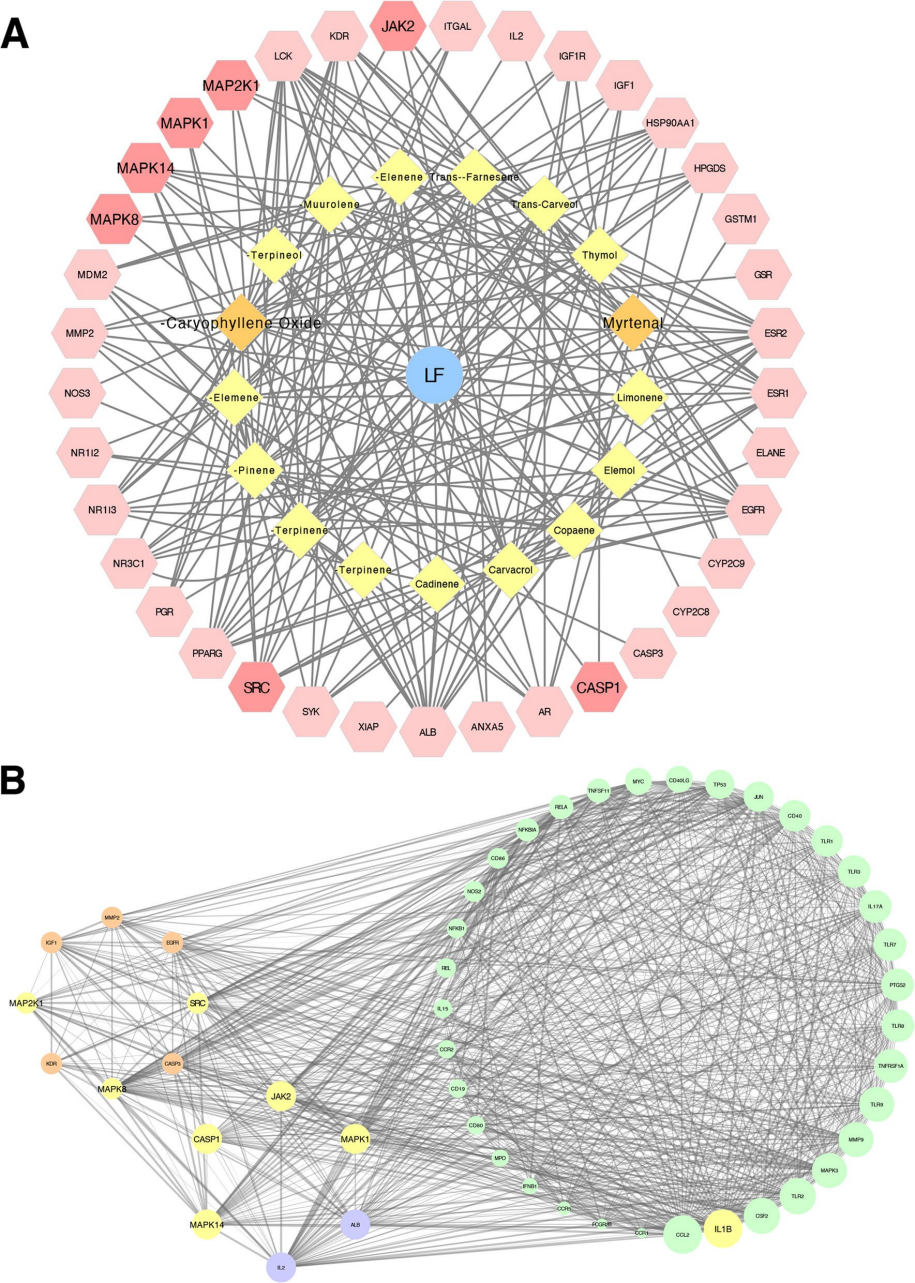
Network pharmacology analysis indicated that many components’ targets were closely related to NLRP3 inflammasome activation. **A** Components of petroleum ether extract of LF (PEL) are represented by diamonds, and their potential targets are represented by hexagons. **B** Optimized PPI network between the components’ targets and the therapeutic targets of rheumatoid arthritis (RA). Components’ targets are marked in orange; therapeutic targets of RA are marked in green, with larger size indicating a more important role in the network; common targets are marked in purple; targets related to NLRP3 inflammasome activation are marked in yellow. Protein targets are represented by their gene names

To investigate the active ingredients and potential mechanism of LF in the treatment of RA, 17 components of PEL were collected from the database and literature as mentioned in the Materials and Methods section. The potential targets of these compounds were predicted and optimized, and 37 targets of the components of LF were obtained (Fig. 6A). One hundred forty-six therapeutic targets in RA were also collected. Construction and optimization of the PPI network between the potential targets of the components and the therapeutic targets of RA yielded a main network containing 49 nodes, including 14 component targets and 35 therapeutic targets, of which 6 targets overlapped. (Fig. 6B). It is worth noting that the therapeutic target IL-1β plays an important role in the PPI network. Additionally, many components’ targets, including Src (*SRC*), JAK2, MEK1 (*MAP 2 K1*), ERK2 (*MAPK1*), JNK1 (*MAPK8*), p38 MAPK (*MAPK14*) and caspase-1 (*CASP1*), had close relationships with the activation of the NLRP3 inflammasome pathway [29–34], indicating that inhibition of NLRP3 inflammasome activation is an important mechanism underlying the anti-arthritis effect of LF.

### Molecular docking

**Fig. 7:**
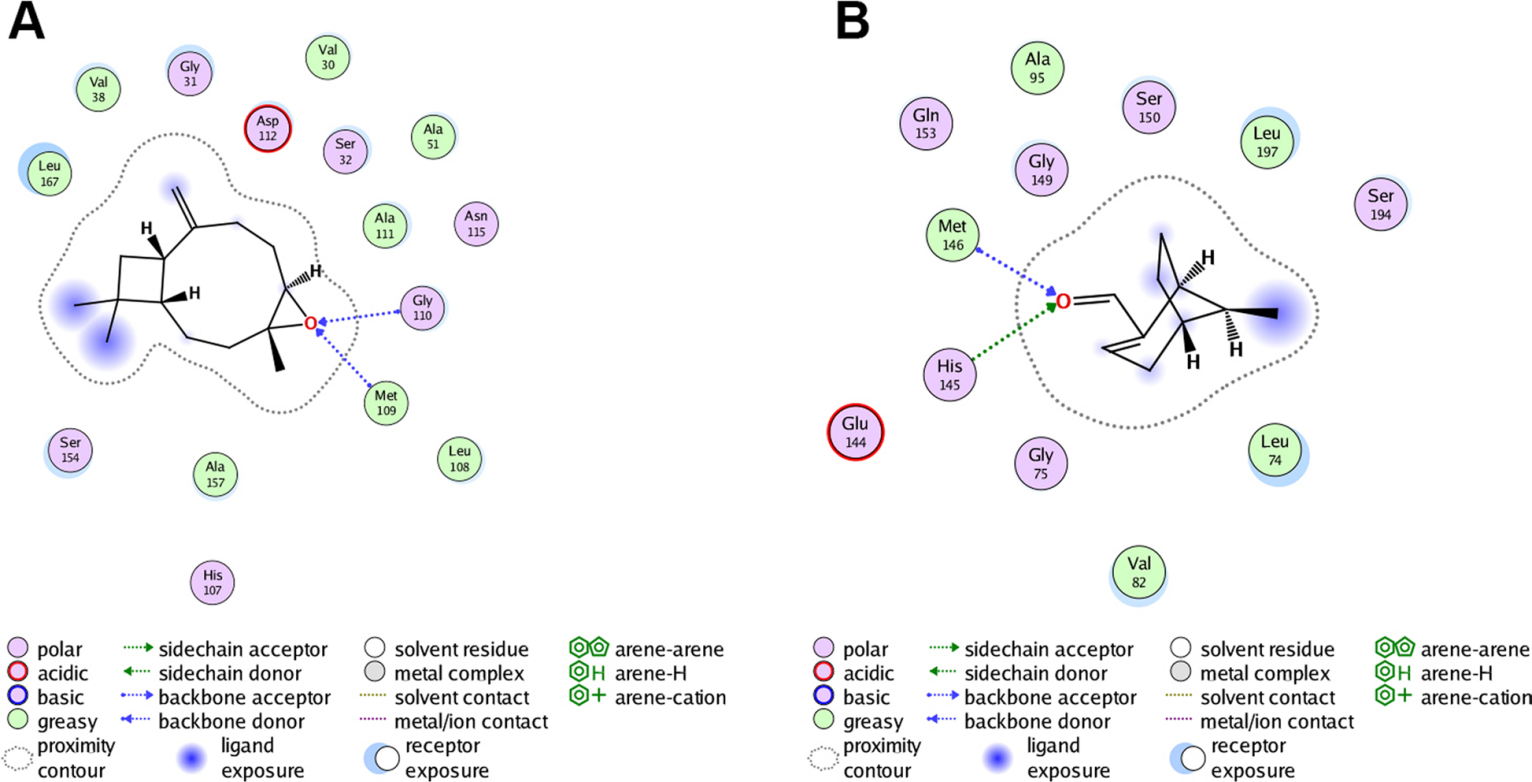
Binding interaction between β-caryophyllene oxide and p38 MAPK, and myrtenal and MEK1. **A** β-caryophyllene oxide can form hydrogen bonds with Met109 and Gly110 in the hinge region of p38 MAPK. **B** Myrtenal can bind with His145 and Met146 in the ATP-binding pocket of MEK1

According to the network pharmacology analysis, 8 of the 14 components’ targets in the PPI network were protein kinases (Fig. 6B). As protein kinases play important roles in the pathogenesis of RA, and small-molecule kinase inhibitors have substantially improved clinical outcomes [35], we used the 17 components of PEL to dock with the 8 protein kinases targets including Src, JAK2, MEK1, ERK2, JNK1, p38 MAPK, EGFR and KDR. We found that these components could bind with protein kinase targets, functioning as inhibitors. Among the 17 components, myrtenal and β-caryophyllene oxide exhibited better binding affinity with the targets. Moreover, among the 8 targets, p38 MAPK had highest binding affinity with β-caryophyllene oxide, whereas MEK1 displayed the highest binding affinity for myrtenal. According to the molecular docking results, β-caryophyllene oxide could form hydrogen bonds with Met109 and Gly110 at the hinge region of p38 MAPK (Fig. 7A), and myrtenal could bind with His145 and Met146 at the ATP-binding pocket of MEK1 (Fig. 7B), indicating they were potential inhibitors of the targets. Both components could also bind with caspase-1, one of the three components of the NLRP3 inflammasome, as its inhibitors. Therefore, myrtenal and β-caryophyllene oxide could be the active ingredients of LF that mediate its effects on arthritis symptoms.

### Myrtenal and β-caryophyllene oxide in PEL inhibited IL-1β and TNF-α production in RA-FLS

**Fig. 8:**
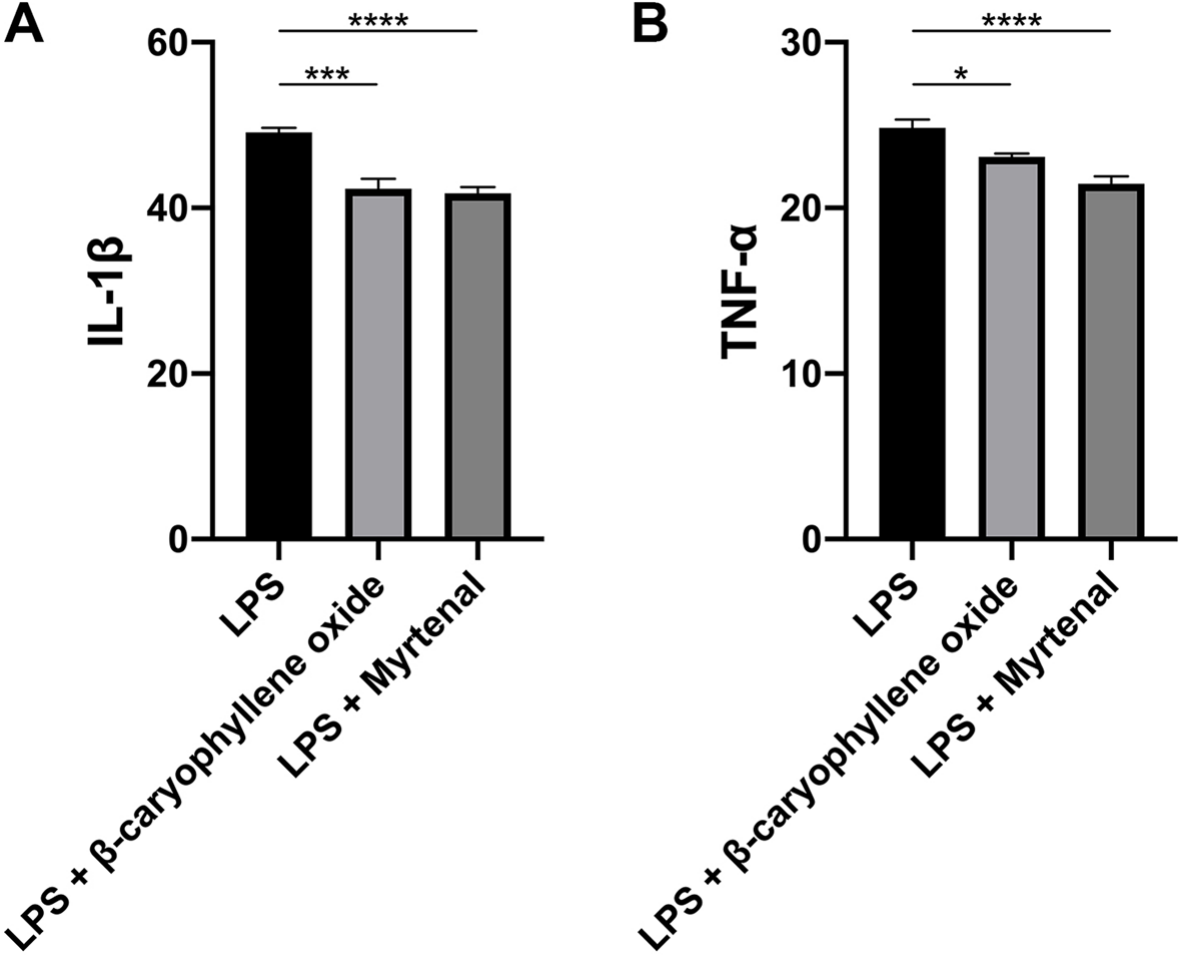
The production of IL-1β and TNF-α in human rheumatoid arthritis fibroblast-like synovial cells (RA-FLS). ELISA was used to quantify the IL-1β and TNF-α secretion. Myrtenal and β-caryophyllene oxide could significantly reduce IL-1β production. The production of TNF-α was also significantly decreased after treatment with myrtenal and β-caryophyllene oxide. Data are recorded as mean ± SEM. Differences were assessed by Dunnett’s multiple comparisons test and one-way ANOVA, *P < 0.05 vs MC, ***P < 0.001 vs MC, ****P < 0.0001 vs MC. (*n* = 6)

To verify if myrtenal and β-caryophyllene oxide are active ingredients of LF in the treatment of RA, we used the RA-FLS model to evaluate the inhibitory effects of myrtenal and β-caryophyllene oxide on the production of IL-1β and TNF-α [36–38]. The results showed that both myrtenal and β-caryophyllene oxide could significantly reduce IL-1β production in RA-FLS. The production of TNF-α was also significantly decreased after treatment with myrtenal and β-caryophyllene oxide (Fig. 8).

### Myrtenal and β-caryophyllene oxide in PEL inhibited activation of the NLRP3 inflammasome pathway in vitro

**Fig. 9:**
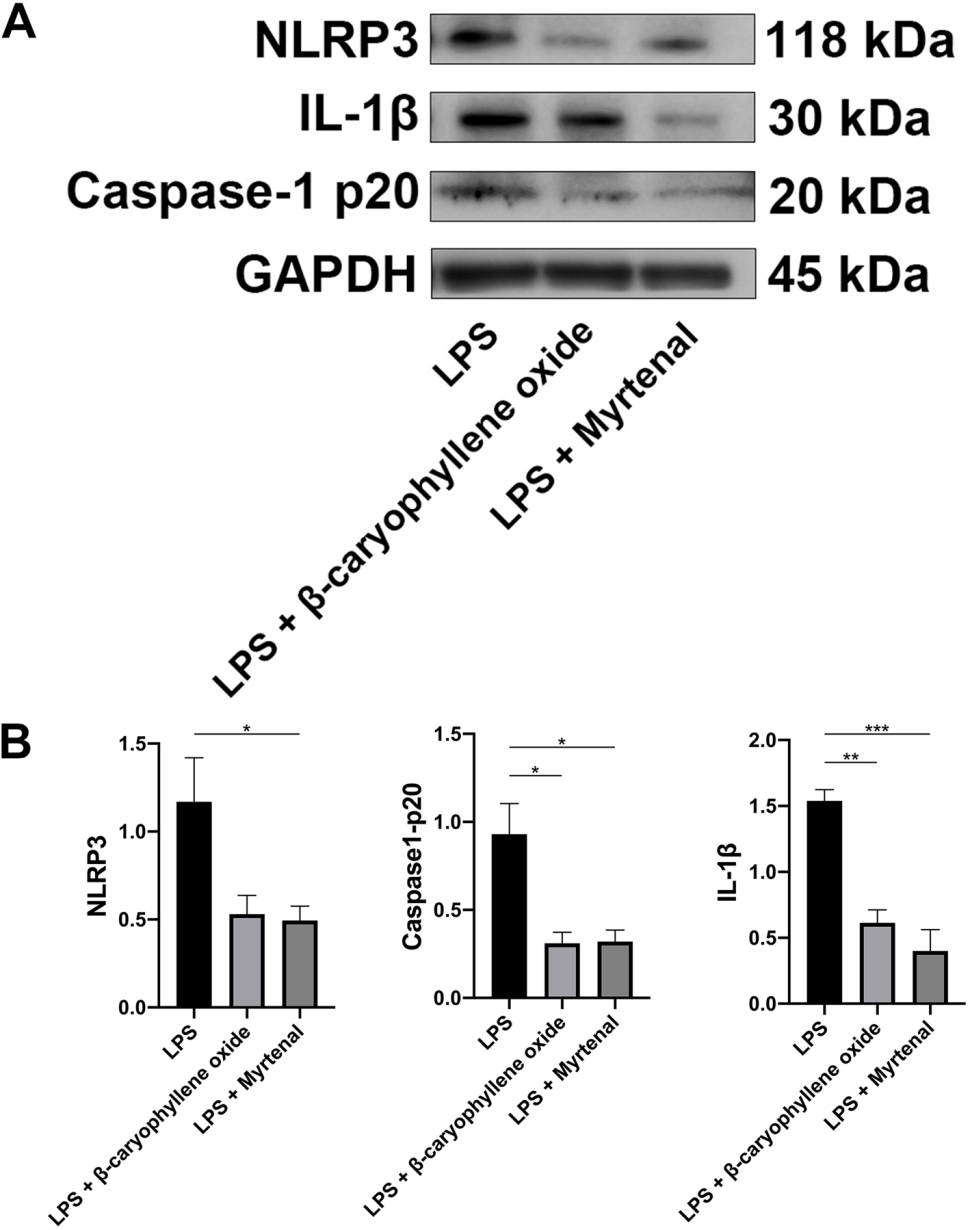
The NLRP3 inflammasome pathway was activated in human rheumatoid arthritis fibroblast-like synovial cells (RA-FLS). The expression of NLRP3, caspase-1 p20 and IL-1β in RA-FLS were significantly reduced after treating with myrtenal. β-caryophyllene oxide showed inhibitory effects on the expression of caspase-1 p20 and IL-1β, but not NLRP3. Data were recorded as mean ± SEM. Differences were assessed by Dunnett’s multiple comparisons test and one-way ANOVA, *P < 0.05 vs MC, **P < 0.01 vs MC, ***P < 0.001 vs MC. (*n* = 3)

To further verify the inhibitory effect of myrtenal and β-caryophyllene oxide on the activation of the NLRP3 inflammasome pathway, we evaluated the expression of NLRP3, caspase-1 p20 and IL-1β in RA-FLS using Western blot. Results showed that myrtenal treatment could significantly suppress the expression of NLRP3, IL-1β and caspase-1 p20. β-caryophyllene oxide treatment could inhibit the expression of caspase-1 p20 and IL-1β, but had no effect on the expression of NLRP3 (Fig. 9).

## Discussion

In Traditional Chinese Medicine, LF has been used to treat joint pain and some gynecological diseases [17]. As joint pain is a common symptom of RA, in this study we explored the anti-RA potential of LF using an AIA rat model. The results showed that LF could alleviate paw swelling, bone destruction, synovial inflammation and cartilage erosion in AIA rats, and the efficacy of PEL was superior to that of EEL and WEL. Triterpenoids are generally considered the main components of LF; however, according to our previous study, triterpenoids exist in EEL and not PEL [39]. The components of PEL are monoterpenoids and sesquiterpenoids (Fig. 5A). Our lab is currently investigating the bioactive chemical ingredients of LF, and in a previous study we investigated the anti-breast cancer effects and mechanisms of action of the triterpenoids in LF [40]. This study supports the traditional utility of LF in the treatment of gynecological diseases. However, this study showed that monoterpenoids and sesquiterpenoids play a more important role than triterpenoids in the anti-RA effect of LF. According to some Traditional Chinese Medicine literature, such as *Bencao Gangmu Shiyi* (1765 AD), LF was burned and the resulting smoke inhaled to treat joint pain, indicating that the active ingredients are volatile components [41].

**Fig. 10:**
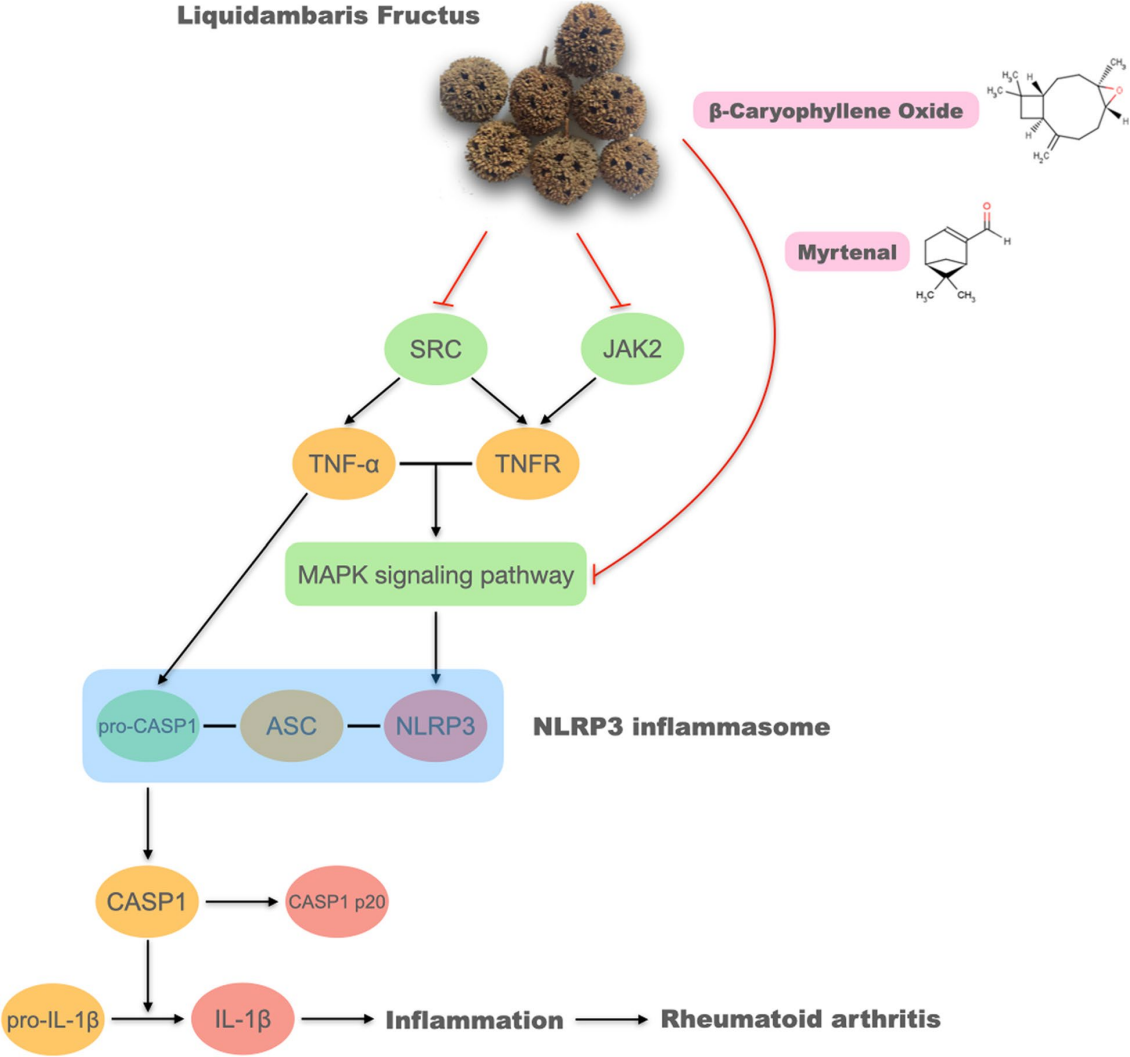
The predicted mechanism of the anti-RA effect of LF upon selected key targets

As one of the most important multimeric protein complexes, NLRP3 inflammasome activation leads to increased cleavage of caspase-1 and downstream IL-1β production, and plays a pathological role in many diseases including diabetes, atherosclerosis and cardiovascular diseases [42–44]. Recently, studies have revealed the significance of the NLRP3 inflammasome in innate and adaptive immune responses, and activation of the NLRP3 inflammasome pathway has been implicated in many autoimmune diseases, such as systemic lupus erythematosus, systemic sclerosis, inflammatory bowel disease and RA [45, 46]. It has been reported that the NLRP3 inflammasome is activated in the synovium of RA mice, and that treatment with a selective NLRP3 inhibitor can reduce the symptoms of arthritis and cartilage destruction [7–9]. Therefore, in this study we evaluated the effects of LF on the expression of IL-1β, caspase-1 p20 and NLRP3 in the ankle joint synovial tissue of AIA rats, and quantified the serum levels of inflammatory cytokines IL-1β and TNF-α. Among the 3 extracts, PEL showed the strongest inhibition on the activation of the NLRP3 inflammasome pathway, which was consistent with the morphological and histopathological results. In addition, network pharmacology analysis also showed that many potential targets of components of PEL, including MEK1, ERK2, JNK1, p38 MAPK, Src, JAK2 and caspase-1, were closely related to the NLRP3 inflammasome pathway. The ERK signaling pathway is an important upstream regulator of the NLRP3 inflammasome. p38 MAPK plays an important role in proinflammatory cytokine synthesis. It has been reported that the activation of p38 MAPK is required for NLRP3-mediated IL-1β secretion in cultured human monocyte - derived macrophages [31]. It has also been reported that inhibition of ERK strongly suppresses NLRP3 expression both in 3 T3-L1 adipocytes induced by TNF-α and in LS14 preadipocytes induced by calcium-sensing receptors [32]. MEK1 activates ERK and functions specifically in the ERK cascade, acting as an essential component of MAPK signal transduction. It has been reported that when THP-1 macrophages are stimulated with oxidized low-density lipoprotein, the MEK/ERK pathway is activated and the expression of NLRP3 inflammasome-related proteins is upregulated [33]. Conversely, MEK inhibitors have been shown to decrease TNF-α induced NLRP3 expression in 3 T3-L1 cells [32]. Taken together, this evidence indicates that components of PEL probably suppress NLRP3 expression by inhibiting the MAPK signal transduction pathway, which might play a major role in the anti-RA effect of LF (Fig. 10). Other components’ targets of LF, including Src and JAK2, are also important regulators of the NLRP3 inflammasome. Cellular responses to TNF are initiated by the TNF receptor [47]. It has been reported that the type 1 TNF receptor (TNFR1) could form complexes with Src or JAK2, and then play a role in activating p38 MAPK, JNK1, Akt and NF-κB [30]. Activation of TNFR1 increased the IL-1β expression in THP-1 macrophages, which could be impeded by inhibitors of Src and JAK2 [30]. Moreover, Src can also activate TNF-α converting enzyme and then lead to increased TNF-α release and p38 MAPK activation in myogenic precursor cells [29]. These lines of evidence suggest that components of PEL are likely to exert their inhibitory effects on caspase-1 expression by inhibiting Src and JAK2, resulting in inhibition of the TNF signaling pathway. After the NLRP3/ASC/pro-caspase-1 protein complex assembles, pro caspase-1 self-cleaves into the activated form, and the activated caspase-1 releases IL-1β precursors into the extracellular cleavage activation. These predicted mechanisms of the anti-RA effect of LF are shown in Fig. 10.

The molecular docking study showed that components of PEL could bind with protein targets including MEK1, ERK2, JNK1, p38 MAPK, Src, JAK2 and caspase-1 as their inhibitors. Among the components, myrtenal and β-caryophyllene oxide exhibited superior binding affinity with these targets, especially MEK1 and p38 MAPK. Since fibroblast-like synoviocytes in the synovial intimal lining play a key role in the pathogenesis of RA by producing cytokines that perpetuate inflammation and proteases that contribute to cartilage destruction [36], they were used to verify the anti-RA potential of myrtenal and β-caryophyllene oxide. Our results showed that these two components could suppress the release of IL-1β and TNF-α and the expression of NLRP3 and caspase-1 p20, similar to the effect of PEL, indicating that they were the active ingredients of LF in the treatment of RA. Given the critical function of NLRP3 activation and induction of IL-1β in the pathogenesis of diseases such as gout, these mechanisms of action suggest that LF could have utility in other diseases as well [48–50].

## Conclusions

PEL can alleviate paw swelling, bone destruction, synovial inflammation and cartilage erosion in AIA rats. These effects are associated with inhibition of the NLRP3 inflammasome activation. Inhibition of the upstream MAPK signaling pathway and the TNF signaling pathway is also likely to be involved. Two components, myrtenal and β-caryophyllene oxide, screened from PEL, showed a similar inhibitory effect on the release of IL-1β and TNF-α and the expression of NLRP3 and caspase-1 in RA-FLS, indicating they might be the major active ingredients of LF in the treatment of RA.

## Supplementary Information

**Additional file 1.**

## Data Availability

The datasets used and analysed during the current study are available from the corresponding author on reasonable request.

## Abbreviations

LF: Liquidambaris Fructus
AIA: Adjuvant-induced arthritis
RA: Rheumatoid arthritis
FLS: Fibroblast-like synovial cells
NLRP3: Nucleotide-binding, oligomerization domain (NOD)-like receptor family, pyrin domain containing 3
TNF: Anti-tumour necrosis factor
PAMP: Pathogen-related molecular patterns
DAMP: Damage-related molecular patterns
HPLC: High performance liquid chromatography
PEL: Petroleum ether extract of LF, EEL, ethyl acetate extract of LF and WEL, water extract of LF
SPF: Specific Pathogen Free
CFA: Freund’s complete adjuvant
Micro-CT: Micro-Computed Tomography
HE: Hematoxylin-eosin
ELISA: Enzyme-linked immunosorbent assay
SDS-PAGE: Sodium dodecyl sulfate-polyacrylamide gel electrophoresis
PVDF: Polyvinylidene fluoride
GAPDH: Glyceraldehyde phosphate phosphate dehydrogenase
HRP: Horseradish peroxidase
ECL: Enhanced chemiluminescence
PPI: Protein-protein interaction
LPS: Lipopolysaccharide
