# Supplementary material for: Myrtenal and β-caryophyllene oxide screened from Liquidambaris Fructus suppress NLRP3 inflammasome components in rheumatoid arthritis
Source: BMC Complement Med Ther. 2021 Sep 28;21:242. doi: 10.1186/s12906-021-03410-2 (PMC8480017; doi:10.1186/s12906-021-03410-2)
Supplement: Supplementary file 1 — Additional file 1. [file 12906_2021_3410_MOESM1_ESM.zip › statement about ARRIVE guidelines.docx]

The study about “Myrtenal and β-caryophyllene oxide screened from Liquidambaris Fructus suppress NLRP3 inflammasome components in rheumatoid arthritis” was carried out in compliance with the ARRIVE guidelines.
